# Case report of cheilitis granulomatosa and joint complaints as presentation of Crohn’s disease

**DOI:** 10.1007/s12328-016-0641-z

**Published:** 2016-03-26

**Authors:** Daniël R. Hoekman, Joris J. T. H. Roelofs, Joost van Schuppen, Dieneke Schonenberg-Meinema, Geert R. D’Haens, Marc A. Benninga

**Affiliations:** Department of Pediatric Gastroenterology and Nutrition, Academic Medical Center, Meibergdreef 9, 1105 AZ Amsterdam, The Netherlands; Department of Pathology, Academic Medical Center, Amsterdam, The Netherlands; Department of Pediatric Radiology, Academic Medical Center, Amsterdam, The Netherlands; Department of Pediatric Rheumatology and Immunology, Academic Medical Center, Amsterdam, The Netherlands; Department of Gastroenterology and Hepatology, Academic Medical Center, Amsterdam, The Netherlands

**Keywords:** Crohn’s disease, Orofacial granulomatosis, Cheilitis granulomatosa, Inflammatory bowel disease, Lip swelling

## Abstract

Cheilitis granulomatosa is characterized by granulomatous lip swelling. We report a case of a 13-year-old girl who presented with orofacial swelling and arthralgia, who eventually was diagnosed with Crohn’s disease, which was successfully treated with infliximab and azathioprine combination therapy. Recurrent or persistent orofacial swelling should prompt consideration of cheilitis granulomatosa, and further diagnostic evaluation to exclude the presence of Crohn’s disease seems warranted.

## Introduction

Cheilitis granulomatosa is a rare clinical entity characterized by swelling of one or both lips caused by granulomatous inflammation, initially described by Miescher in 1945. It may occur as an isolated phenomenon, or as a manifestation of systemic disease. Orofacial granulomatosis (OFG) is an umbrella term used to describe patients with granulomatous oral lesions with no evidence of a systemic granulomatous condition. Orofacial involvement in patients with gastrointestinal Crohn’s disease (CD) is classified as oral CD. OFG may precede a diagnosis of CD.

We report a case of an adolescent patient who presented with a history of orofacial swelling and transient arthralgia, who was eventually diagnosed with CD.

## Case report

**Fig. 1:**
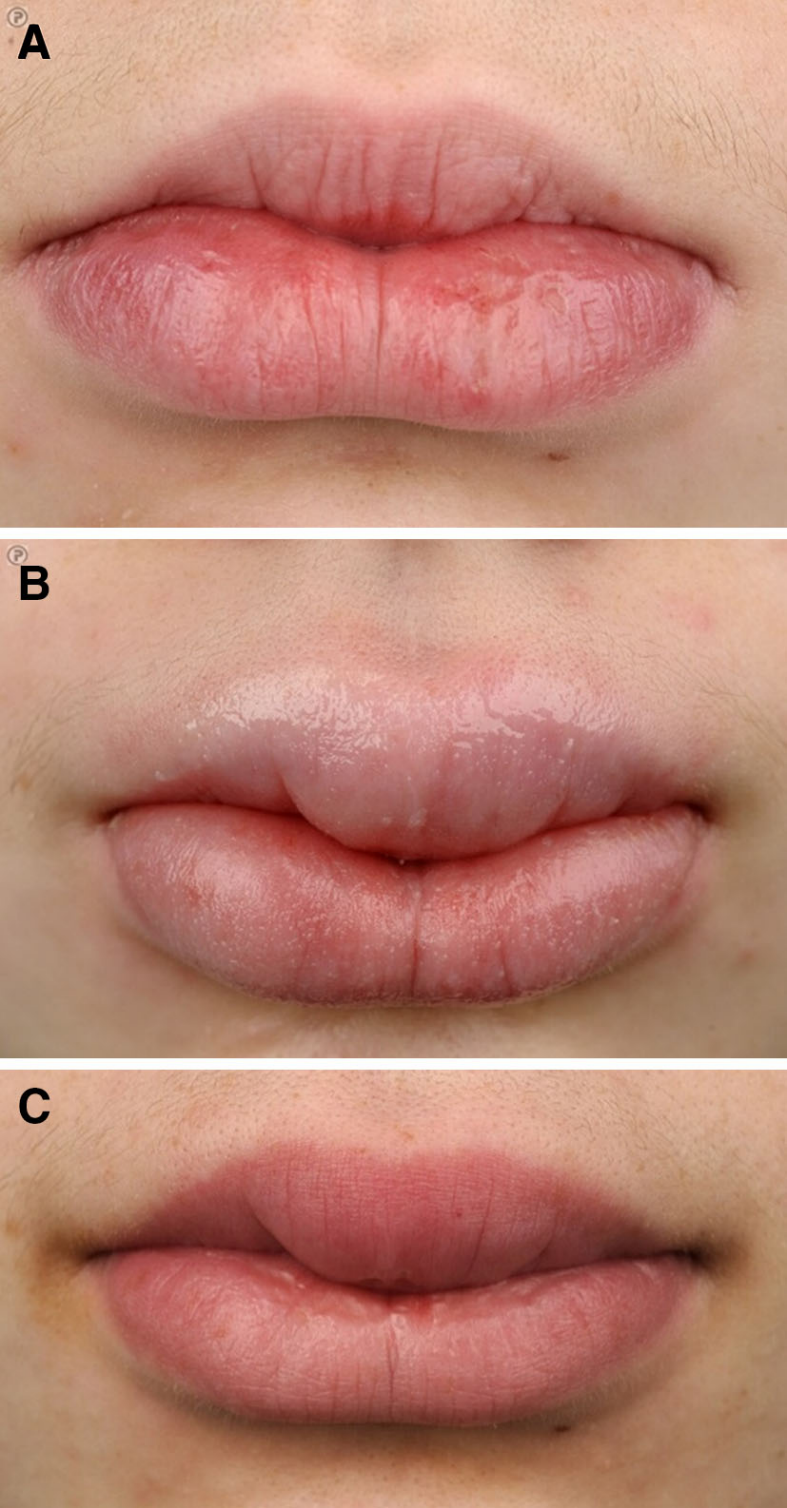
**a** Patient presentation 5 months after initial presentation; **b** around the start of gastrointestinal symptoms; and **c** 1 year after initiation of IFX and azathioprine combination therapy

A 13-year-old, white, female patient with a history of adenotonsillectomy for recurrent tonsillitis initially presented in May 2011 with ankle pain for 6 months, which was associated with transient episodes of joint swelling. There was no history of trauma. She also had frequent sore throats without evidence of infectious pharyngitis. She had no history of recurrent oral aphthous lesions. On initial physical examination, there were no signs of arthritis, although the medial side of the ankle was painful on palpation. Furthermore, marked swelling of the lower lip was observed (Fig. 1a). Laboratory evaluation revealed an elevated C-reactive protein (CRP 28 mg/L), erythrocyte sedimentation rate (ESR 60 mm/h), anti-streptolysin O titer (11,300 U/mL) and anti-DNase B titer (5600 U/mL). Cardiologic evaluation showed no signs of rheumatic fever. Magnetic resonance imaging of the ankle joint showed no abnormalities. Orthopedic evaluation revealed pes planovalgus, and inlays were prescribed but did not yield improvement. Neurological evaluation showed no abnormalities. The swelling of the lip was interpreted as angioedema and/or the result of lip biting caused by psychological distress. Because of a suspected disturbed pain perception, the patient was treated with physical therapy, which resulted in improvement of the joint pain. However, the lip swelling worsened progressively and both ESR and CRP remained elevated. The high streptococcal antibody titer declined to a normal value in a few months. Serum angiotensin converting enzyme was not elevated.

**Fig. 2:**
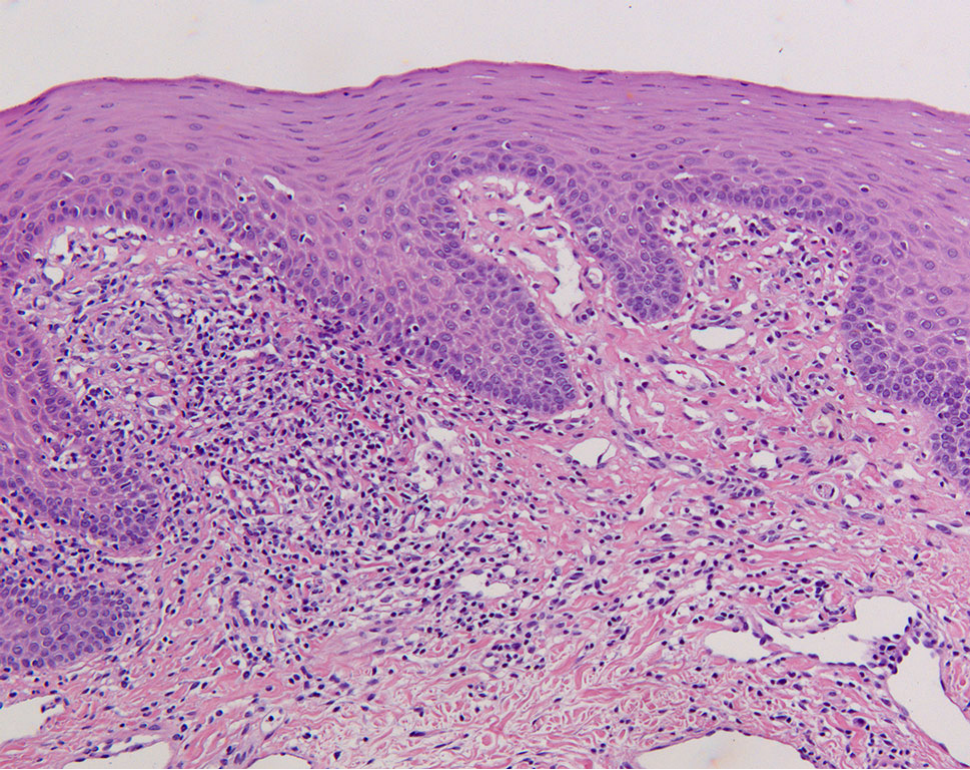
Low-power view microphotograph of labial biopsy showing non-characteristic signs of chronic inflammation. No granulomas were found. Original magnification 100×

**Fig. 3:**
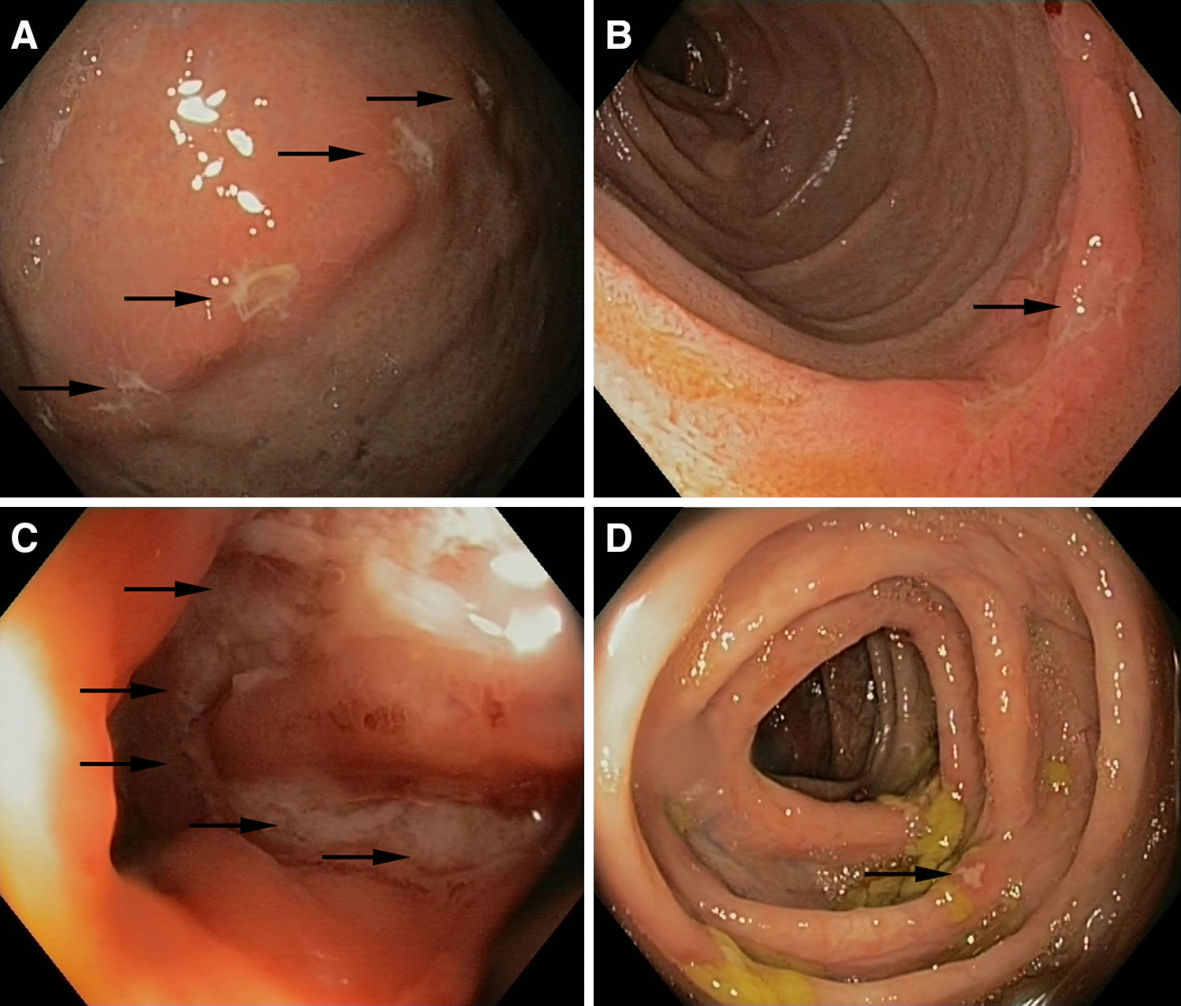
Macroscopic findings at upper and lower endoscopy. **a** Stomach with diffuse aphthous ulceration (*arrows*), erythema and edema of the mucosa; **b** duodenum with aphthous ulceration (*arrows*) and erythema and edema of the mucosa; **c** Terminal ileum with deep, longitudinal ulceration (*arrows*) and erythema and edema of the mucosa; **d** Colon with aphthous ulceration (*arrows*) after suboptimal bowel preparation

**Fig. 4:**
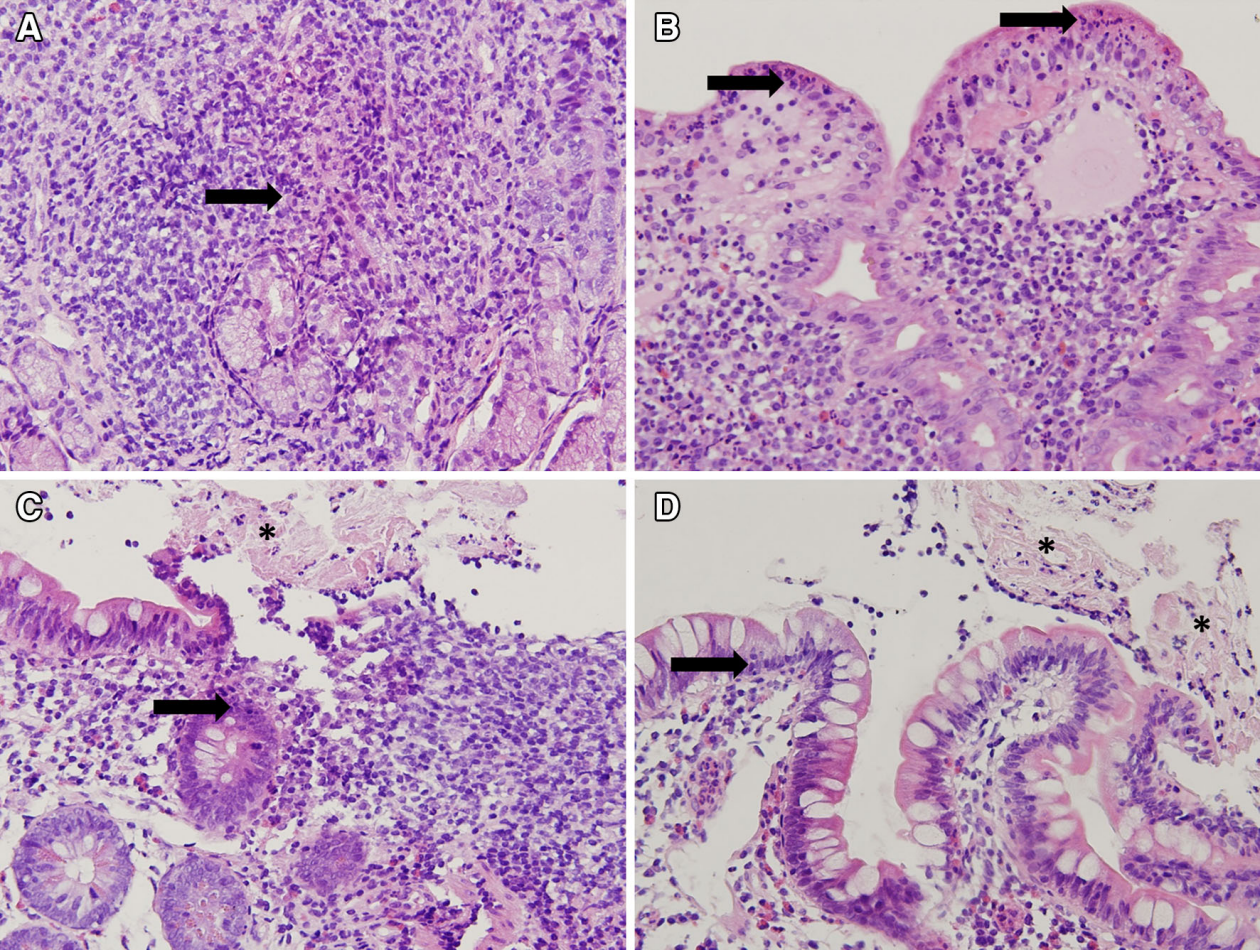
Microphotographs of stomach (**a**), duodenum (**b**), and ileum (**c**, **d**) biopsies. Stomach and duodenum mucosa shows active inflammation characterized by epithelial invasion by neutrophil granulocytes (*arrows*). Ileum tissue also showed neutrophil invasion (*arrows*). In addition, there were signs of ulceration characterized by fibrin deposits (*asterisks*) admixed with inflammatory cells on the mucosal surface. Although no granulomas were found, these findings are consistent with Crohn’s disease. Original magnification 20×

**Fig. 5:**
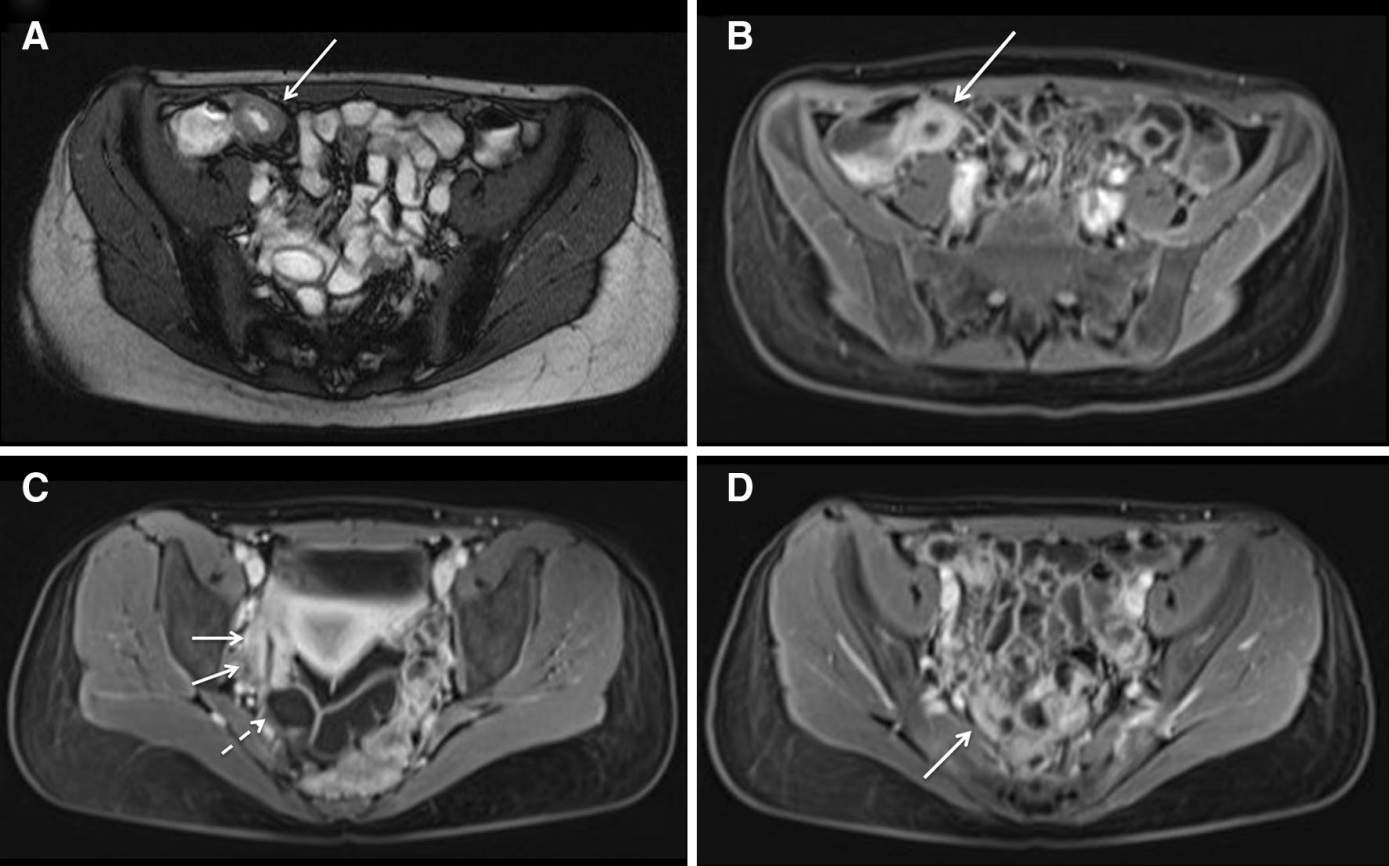
Magnetic resonance images: **a** Axial T2 trufi of the pelvis and **b** axial T1 post contrast at the same level, revealing layered bowel wall thickening with post-contrast enhancement of the terminal ileum (*arrow*). **c** Axial T1 post contrast shows a skip lesion with a length of 6 cm, proximal in the ileum, with bowel wall thickening and post-contrast enhancement (*arrow*); also, some prestenotic dilatation of the bowel is seen (*dashed arrow*). **d** Axial T1 post contrast shows a second skip lesion, with a length of 2 cm, more proximal in the ileum (*arrow*)

Because of persistent swelling of the lip (Fig. 1b—February 2013), the patient was evaluated by an expert in oral pathology, who made a clinical diagnosis of orofacial granulomatosis (cheilitis granulomatosa). Histopathological evaluation of a biopsy of a swollen part of the lip showed a mononuclear inflammatory infiltrate and no granulomas (Fig. 2—June 2013). Topical clobetasol was initiated, with little effect. A few months later, the patient developed abdominal pain, accompanied by altered bowel habits and weight loss. Physical examination showed abdominal tenderness, but no other abnormalities; specifically, no perianal abnormalities. Laboratory evaluation revealed elevated levels of CRP (31.0 mg/L) and fecal calprotectin (>1800 µg/g). Subsequent gastroscopy and ileocolonoscopy performed in August 2013 revealed multiple large, longitudinal ulcers in the terminal ileum and multiple aphthous lesions throughout the colon and in the duodenum and stomach (Fig. 3). Biopsies showed inflammatory changes compatible with CD (Fig. 4). Magnetic resonance imaging showed several sites of wall thickening, with pathologic contrast enhancement in the terminal ileum and more proximal ileum, and prestenotic dilatation in one segment (Fig. 5). The Mantoux-test and a chest X-ray showed no signs of tuberculosis. Thus, a diagnosis of CD could be confirmed according to the Porto criteria [1]. A Pediatric Crohn’s Disease Activity Index (PCDAI) score of 32.5 at the time of diagnosis indicated moderate to severe CD [2].

Exclusive enteral feeding combined with azathioprine (125 mg once daily, i.e., 2.5 mg/kg) was started, without clinical response. Four weeks later, induction treatment of infliximab (5 mg/kg at week 0, 2 and 6) was started, to which the patient responded rapidly. The abdominal pain subsided, the lip swelling decreased, laboratory values normalized, and the patient started gaining weight (weight for length: −0.3 standard deviation (SD) to +0.5 SD in 3 months).

The patient is currently receiving infliximab (5 mg/kg every 8 weeks) and azathioprine (125 mg once daily) as combination maintenance therapy for over 1 year and is in clinical remission (according to a PCDAI score of 5). Biochemical markers of inflammation decreased markedly but did not normalize completely (CRP 3.8 mg/L, fecal calprotectin 272 µg/g). Although less pronounced, the patient’s lips have remained fairly swollen until now (Fig. 1c). To date, no repeat endoscopy or imaging has been performed.

## Discussion

This patient presented initially with cheilitis granulomatosa and arthralgia, which in retrospect were likely to be extraintestinal manifestations of CD. At presentation, however, there were no symptoms or signs (e.g., diarrhea, abdominal pain, perianal fistula) of intestinal disease. Therefore, no gastrointestinal evaluation was performed, and a diagnosis of orofacial granulomatosis (OFG) was made. In retrospect, earlier extensive diagnostic evaluation could possibly have led to an earlier diagnosis of CD.

OFG is a clinical diagnosis, based on the presence of recurrent or persistent orofacial swelling. The lips are the most commonly affected area [3]. Histopathological evaluation typically shows non-caseating granulomas, although this is not always the case, as in our patient [4]. Clinical and laboratory evaluation may be required to exclude systemic conditions that can result in a clinical presentation similar to OFG, including CD, tuberculosis, and sarcoidosis (Table 1) [5].

**Table 1:** The differential diagnosis of orofacial granulomatosis

Disease	Differentiating features from OFG
Crohn’s disease	Gastrointestinal (usually ileal/rectal) disease. Oro-cutaneous fistulas may (rarely) occur in CD. Ulceration, and buccal-sulcal involvement occurs more frequently in CD
Sarcoidosis (usually chronic)	Affected patients may also have pulmonary, cutaneous, lacrimal, salivary neurological and/or skeletal features of sarcoidosis
Allergic angioedema	Non-pitting edema of the lips, tongue, pharynx and face. Can be a feature of anaphylaxis. There may be an identifiable precipitant and patients may have a history of atopic disease (allergic rhinitis, asthma, atopic eczema or drug allergies)
Miescher’s cheilitis (Schuermann’s granulomatous cheilitis)	Manifests as labial enlargement and has similar histopathology to OFG
Melkersson–Rosenthal syndrome	Manifests as labial enlargement, fissuring of the tongue, and lower motor neuron facial nerve palsy—a variant of OFG
Cheilitis glandularis	Labial enlargement with ulcers. Unknown cause (may be trauma/ill-defined infection). There is mild acute and chronic inflammation (without granulomas) within the minor salivary glands of the lip
Tuberculosis	Rarely affects the lips. Manifests as localized swelling and ulcers. Usually arises in immigrant groups and HIV-infected individuals. Usually contains caseating granulomas

Adapted from Grave et al. [5]

OFG may precede a diagnosis of CD. Patients presenting with OFG during childhood are more likely to develop CD, than patients who present with OFG during adulthood [3]. A recent systematic review showed that CD was diagnosed in 40 % of children with OFG, either at the time of presentation of OFG, or during the following months or years [6]. The reported prevalence of oral lesions in established CD has varied widely, from 0.5 to 48 % [7]. Clinical and laboratory evaluation may help to distinguish OFG from oral CD. Oral ulceration and involvement within the buccal sulcus are more common in patients with oral CD, as well as an elevated CRP and abnormal full blood count (especially anemia) [3]. Previous studies have shown that a large proportion (37–54 %) of patients with OFG without other gastrointestinal symptoms have evidence of intestinal inflammation on endoscopy and/or histology [8]. Whether this always reflects CD is unknown, since the clinical relevance of this finding remains to be established.

Given the high likelihood of (developing) CD in pediatric-onset OFG, it has been suggested that in children with OFG, ileocolonoscopy may be indicated even in the absence of intestinal symptoms [3]. In our opinion, given the availability of fecal calprotectin as a non-invasive, sensitive screening tool for suspected inflammatory bowel disease, we suggest that all patients with a suspected diagnosis of OFG undergo biochemical testing, as well as subsequent endoscopy if biochemical markers of intestinal inflammation are elevated. To our knowledge, only one report of two cases touched on the discriminating value of calprotectin in suspected OFG; only the patient with gastrointestinal involvement had an elevated fecal calprotectin [9].

No reliable data on the epidemiology of OFG are available. The pathophysiology is unknown, although various mechanisms have been proposed, including hypersensitivity to food substances or additives or dental materials, the presence of various micro-organisms, and genetic factors [10].

Treatment of OFG depends on whether an underlying systemic cause has been identified. Many different treatment strategies for oral CD have been reported, including local and systemic corticosteroids, anti-TNF-agents, and immunomodulators. There are no data on the comparative efficacy of different treatment options, since no comparative studies have been performed. The presented patient failed to respond to exclusive enteral nutrition. According to a step-up algorithm, remission induction with systemic steroids would likely be the next step. However, since the patient’s oral symptoms previously failed to respond to topical class 4 corticosteroids, infliximab was initiated. This resulted in a rapid response, which has been reported previously [11].

In our patient, the lips remained fairly swollen despite successful treatment of gastrointestinal symptoms. In oral CD, oral disease activity often does not parallel disease activity elsewhere in the intestine [12]. Furthermore, symptoms of OFG or oral CD persist in a substantial proportion of patients. In a review of 49 patients with OFG, after a mean follow-up of 2.9 years, only 40 % achieved complete resolution of symptoms despite treatment [13]. Also, in a cohort of 24 children with oral CD, after a mean follow-up of 55 months, 29 % had persisting or recurrent symptoms of oral CD [12]. After some years, the swelling may slowly regress. In severely disfiguring cases, cheiloplasty may be indicated. This should only be performed once the disease is brought into a quiescent phase [4].

In conclusion, recurrent or persistent orofacial swelling should prompt consideration of OFG, and further diagnostic evaluation to confirm or exclude the presence of CD (e.g., fecal calprotectin) seems warranted, especially in adolescents.

## Abbreviations

CD: Crohn’s disease
OFG: Orofacial granulomatosis
